# The association between blood groups and maxillofacial deformities

**DOI:** 10.4103/0970-0358.44921

**Published:** 2008

**Authors:** Rasoul Gheisari, Mehdi Ghoreishian, Bijan Movahedian, Amrolah Roozbehi

**Affiliations:** Department of Oral and Maxillofacial Surgery, Alzahra hospital, Isfahan University of Medical Sciences, Isfahan, Iran; 1 Department of Anatomy, Yasuj University of Medical Sciences, Yasuj, Iran

**Keywords:** ABO blood grouping, association, blood group type, maxillofacial deformities

## Abstract

**Background:**

Blood group is a genetic characteristic which is associated with some diseases and deformities. Multifactorial characteristics of facial development make it difficult to predict a genetic pattern in a specific maxillofacial deformity, but epidemiological evaluations can reveal relationships between such deformities and some genetic characteristics or accompanied diseases, and this will help to recognise and treat them. The aim of this study is evaluation of the relationship between blood groups and maxillofacial deformities.

**Materials and Methods:**

In this study, blood groups of 190 patients with maxillofacial deformities who had had orthognathic surgery in Alzahra hospital, Isfahan, were compared with the general Iranian population.

**Results:**

Among 190 patients, 93 cases (49%) were men and 97 cases (51%) were women. Fifteen cases (8%) were < 20 years old, 130 cases (68%) were 20–30 years old, and the others (45 cases, 24%) were > 30 years old. The blood group distribution in our samples was as follows: blood group O = 76 cases (40%), blood group A = 58 cases (30%), blood group B = 41 cases (22%), and blood group AB = 15 cases (8%). Among these patients, 31 cases (16%) had maxillary deformities and 27 cases (14%) suffered from mandibular deformities while the other 132 cases (70%) had bimaxillary problems. The Chi-square test showed statistically significant differences between the blood group distribution of the patients of this study and the normal Iranian population (*P* < 0.001).

**Conclusion:**

It was shown that among different blood groups; those with blood group B have a greater likelihood of association with maxillofacial deformities. On the other hand, the probability of the association of such deformities was the least with blood group A.

## INTRODUCTION

Epidemiological studies have shown that around 20% of the world's population suffers from some major maxillofacial deformity. In some cases, the severity is so high that it influences their facial proportion; finally, 5% can be considered to have a physical disability.[1] Approximately 12% of the American population has class II malocclusion, whereas class-III occurs in 1% of this society of which 33% are candidates for surgery.[2]

Malocclusion and other deformities in facial skeletal components may be acquired or hereditary. Indeed, genetics plays an important role in maxillofacial deformities. Hereditary patterns such as familial adherence to a prognathic or retrognathic mandible are often seen in patients with such deformities. However, multifactorial characteristics of facial development make it difficult to predict a genetic pattern in a specific maxillofacial deformity, but epidemiological evaluations can reveal relationships between such deformities and some genetic characteristics or accompanied diseases which will help to recognise and treat them.

One of the most important human genetic characteristic is the relationship between the ABO blood group system and some diseases and deformities.[3] The ABO blood group system is the first and the most important system defined in 1901.[4] There are two main antigens, A and B, in the ABO system, present on cell membranes or secreted into the plasma and other fluids of the body. The presence or absence of these antigens results in the four blood groups: A, B, AB, and O. These antigens are present on the 9^th^ chromosome and are inherited co-dominantly.[5]

Due to the lack of information on the association of blood groups with maxillofacial deformities, this study was conducted to fill this lacuna and we hope that our findings are beneficial for future research.

## MATERIALS AND METHODS

This is a cross-sectional study conducted in the Department of Oral and Maxillofacial Surgery of Alzahra hospital, Isfahan. One hundred and ninety cases who had had orthognathic surgery between 2001 and 2006, and for whom complete information about their facial deformity and blood group type recorded as per the ABO system was available, were included in the study. None of them had any systemic disease or congenital syndromes. They were classified into three groups, maxillary, mandibular, and bimaxillary deformities based on preoperative analyses including clinical evaluation, lateral cephalometry, and dental cast studies. The objectives of our treatment in all patients regardless of their sex was to gain the normal class I occlusion and profile.

Statistical analysis of the information obtained was performed using SPSS software (version 11.5) and the Chi-square Test. The differences with *P* < 0.001 were considered statistically significant.

## RESULTS

Among 190 patients, 93 cases (49%) were men and 97 cases (51%) were women. Fifteen cases (8%) were < 20 years old, 130 cases (68%) were 20–30 years old, and the others (45 cases, 24%) were > 30 years old. Our study showed that the incidence of bimaxillary deformities was higher than that of each jaw deformities separately, whereas mandibular deformities were the least prevalent. 31 patients (16%) had maxillary deformities and 27 (14%) suffered from mandibular deformities while the other 132 (70%) had bimaxillary deformities). In addition, the blood group distribution in our study was: blood group O: 76 cases (40%), blood group A: 58 cases (30%), blood group B: 41cases (22%), and blood group AB: 15 cases (8%).

The blood group distribution for patients with maxillofacial deformities in comparison with normal Iranian society is shown in Table 1. Statistically significant differences were found between them (*P* < 0.001) after chi-square analyses.

**Table 1:** Blood group distribution in Iranian society and our study group

*Blood groups*	*Study group (%)*	*Norm of Iran (%)*
A	30	40
B	22	10
AB	8	5
O	40	45
Total	100	100

## DISCUSSION AND CONCLUSION

The aim of this study was to determine if there was a relationship between blood groups and maxillofacial deformities. Our data suggest that there seem to be deformities that occur with a greater incidence in people with a specific blood group.

The blood group distribution in different populations such as, African,[6] Caucasian,[7] England,[8] Iran[9] and the other areas have been studied. The most prevalent blood group was found to be O and the least prevalent one was AB.[10]

Blood groups are considered to be important for the sake of blood transfusion but some studies have illustrated the statistical relationship of blood groups and some specific diseases [Table 2].[11–14] Reid and Hadley have shown the relationship between blood group and congenital cataract in the Asian race.[15,16] Another study demonstrated that *Helicobacter pylori* infection is closely associated with blood groups A and O, which are in agreement with other published data, whereas the blood group AB, smoking, and the male gender function as protective factors against *H. pylori* infection.[17] All these studies included only two aspects, the pathology and the blood type.

**Table 2:** Different blood groups and related diseases

*Blood groups*	*Related diseases*
A	Carcinoma of stomach, salivary gland, colon, rectum, uterus, cervix, bladder--pernicious anemia
O	Duodenal and gastric ulcer, rheumatoid arthritis, von Willebrand disease, typhoid
B	E. coli, urinary tract infection, gonorrhoea
AB	Duodenal ulcer, Streptococcal infection

As mentioned before, the order of incidence of blood groups seen commonly in patients suffering from maxillofacial deformities were O, A, B, and AB. Statistical analysis with the Chi-square test showed that blood groups B and AB have an increased incidence of association with maxillofacial deformities (B > AB) whereas blood groups O and A have a lower incidence of association (A > O).

According to our results in comparison with members of the normal Iranian society, maxillofacial deformities have a greater incidence of association with blood group B due to which it may be expected to see more deformities in this blood group and fewer deformities in blood group A individuals. Further differentiations and other reasons should also be considered, warranting a more comprehensive study.
